# Loss of Hepatic CEACAM1: A Unifying Mechanism Linking Insulin Resistance to Obesity and Non-Alcoholic Fatty Liver Disease

**DOI:** 10.3389/fendo.2017.00008

**Published:** 2017-01-26

**Authors:** Garrett Heinrich, Hilda E. Ghadieh, Simona S. Ghanem, Harrison T. Muturi, Khadijeh Rezaei, Qusai Y. Al-Share, Thomas A. Bowman, Deqiang Zhang, Robert S. Garofalo, Lei Yin, Sonia M. Najjar

**Affiliations:** ^1^Department of Biomedical Sciences, Heritage College of Osteopathic Medicine, Ohio University, Athens, OH, USA; ^2^Heritage College of Osteopathic Medicine, Diabetes Institute, Ohio University, Athens, OH, USA; ^3^Center for Diabetes and Endocrine Research (CeDER), College of Medicine and Life Sciences, University of Toledo, Toledo, OH, USA; ^4^Department of Molecular and Integrative Physiology, University of Michigan Medical School, Ann Arbor, MI, USA; ^5^Yale Cancer Center, Office of Research Affairs, New Haven, CT, USA

**Keywords:** insulin clearance, insulin resistance, lipogenesis, fatty liver oxidation, lipolysis, NAFLD, visceral obesity

## Abstract

The pathogenesis of human non-alcoholic fatty liver disease (NAFLD) remains unclear, in particular in the context of its relationship to insulin resistance and visceral obesity. Work on the carcinoembryonic antigen-related cell adhesion molecule 1 (CEACAM1) in mice has resolved some of the related questions. CEACAM1 promotes insulin clearance by enhancing the rate of uptake of the insulin-receptor complex. It also mediates a negative acute effect of insulin on fatty acid synthase activity. This positions CEACAM1 to coordinate the regulation of insulin and lipid metabolism. Fed a regular chow diet, global null mutation of *Ceacam1* manifest hyperinsulinemia, insulin resistance, obesity, and steatohepatitis. They also develop spontaneous chicken-wire fibrosis, characteristic of non-alcoholic steatohepatitis. Reduction of hepatic CEACAM1 expression plays a significant role in the pathogenesis of diet-induced metabolic abnormalities, as bolstered by the protective effect of hepatic CEACAM1 gain-of-function against the metabolic response to dietary fat. Together, this emphasizes that loss of hepatic CEACAM1 links NAFLD to insulin resistance and obesity.

## Physiologic Regulation of Carcinoembryonic Antigen-Related Cell Adhesion Molecule 1 (CEACAM1)

The CEACAM1 is a transmembrane glycoprotein that undergoes phosphorylation by the insulin receptor tyrosine kinase (1). Among insulin target tissues, CEACAM1 is predominantly expressed in the liver (2). This is consistent with its role in promoting insulin clearance, which occurs mostly in liver and to a lower extent in kidney. Consistent with the important role of the liver in regulating insulin and lipid metabolism, Ceacam1 transcription is coordinately regulated by insulin and fatty acids during fasting–refeeding conditions, with fatty acids at fasting repressing it *via* a mechanism depending on the peroxisome proliferator-activated receptor alpha (PPARα) (3, 4) and insulin inducing it in the first few hours of refeeding (3, 5).

## CEACAM1 Promotes Insulin Clearance and Mediates an Acute Negative Effect of Insulin on Hepatic *De Novo* Lipogenesis

Insulin is released from pancreatic β-cells in a pulsatile manner (6). The acute rise of insulin in the portal vein causes phosphorylation and activation of the insulin receptor tyrosine kinase in the hepatocyte (7, 8). This, in turn, leads to phosphorylation of substrates, including CEACAM1 (1). Upon its phosphorylation, CEACAM1 promotes receptor-mediated insulin uptake into clathrin-coated pits/vesicles of the hepatocyte to be eventually degraded and cleared from the blood (9, 10). This process mediates the rapid extraction of ~50% of secreted insulin through its first pass into the liver.

Internalization of phosphorylated CEACAM1 as part of the insulin-receptor complex leads to its binding to fatty acid synthase (FASN) (11), a key enzyme that catalyzes the conversion of malonyl-CoA to palmitic acid during *de novo* lipogenesis. CEACAM1 association downregulates FASN enzymatic activity and restricts hepatic *de novo* lipogenesis, likely to protect the liver against the potential lipogenic effect of approximately twofold to threefold higher level of insulin in the portal than the systemic circulation (12). Thus, CEACAM1 phosphorylation by the insulin receptor in response to acute rise of insulin constitutes a key mechanism that underlies the maintenance of physiologic insulin levels, at the same time as mediating a suppressive acute effect of insulin on lipogenesis in liver. Combined, this restricts hepatic lipid production under normal physiologic conditions; assigning a major role for CEACAM1 in integrating the regulation of insulin and lipid metabolism in the hepatocyte. Under conditions of hyperinsulinemia, the pulsatility of insulin secretion is compromised (6), limiting insulin signaling in the hepatocyte, including CEACAM1 phosphorylation, and subsequently, the acute negative effect of insulin on FASN activity is removed to contribute to hyperinsulinemia-driven lipogenesis (11). This paradigm emphasizes the contrast between the previously unappreciated suppressive effect of acute insulin pulses on fatty acid synthesis and the well-recognized positive effect of chronically elevated levels of insulin on lipogenic genes’ expression by the coordinated action of sterol regulatory element-binding protein (SREBP1c) (13) and the upstream stimulatory factor 1 (14). Suppression of hepatic FASN activity by pulsatile insulin release proposes to include elevation in *de novo* lipogenesis as a manifest of hepatic insulin resistance in addition to increased hepatic glucose production (*via* glycogenolysis and gluconeogenesis) (8, 15).

## Mutating CEACAM1 in Liver Causes Insulin Resistance and Non-Alcoholic Steatohepatitis (NASH)

Mice with liver-specific inactivation (L-SACC1) or with global null mutation of *Ceacam1* (*Cc1*^−/−^) exhibit impairment in insulin clearance leading to chronic hyperinsulinemia and systemic insulin resistance (owing to downregulation of insulin receptor expression) (16–18). They also exhibit elevated lipid production in liver and redistribution to the white adipose tissue to be stored; thus, contributing to visceral obesity and increased release of free fatty acid (FFA) and adipokines (19).

Mutant Ceacam1 mice also develop inflammation in liver, in part due to the loss of the anti-inflammatory effect of CEACAM1 (20), apoptosis, and oxidative stress. Additionally, they manifest chicken-wire bridging fibrosis, a characteristic feature of NASH, even when fed a standard chow diet, making them rare mouse models of spontaneous fibrosis on the C57BL/6J genetic background. The underlying mechanisms of fibrosis in Ceacam1 mutants are the subject of intense investigations in our laboratories.

## Dietary Fat Reduces Hepatic CEACAM1 Expression in C57BL/6J Mice

In uncomplicated obesity with low-grade insulin resistance, FFA are mobilized from white adipose tissue mainly to the liver to be removed by β-oxidation (21). This is supported by experimental evidence in rodents showing occurrence within few days of the initiation of high-fat intake as a result of dysregulated hypothalamic control in the adipose tissue (22). While this early lipolysis occurs in the absence of insulin resistance in the adipose tissue, the released FFA can rapidly initiate hepatic insulin resistance (23), in part by activating PKCδ-mediated pathways (24). As the nutritional burden persists, hepatic lipotoxicity develops in response to progressively compromised β-oxidation relative to re-esterification. Concomitantly, hepatic insulin resistance progresses into systemic insulin resistance to be manifested in peripheral tissues, including the white adipose tissue with ensuing advancement of a pro-inflammatory state (25).

Recent reports from our laboratories show that high-fat diet progressively reduces hepatic CEACAM1 level in C57BL/6J mice until it reaches >50% after 3 weeks, at which point, insulin clearance is impaired and hyperinsulinemia develops with attendant hepatic insulin resistance and steatohepatitis (26). Consistent with the key role for CEACAM1 in diet-induced insulin resistance and hepatosteatosis, adenoviral-mediated redelivery of wild-type, but not phosphorylation-defective CEACAM1 to the liver, completely reverses these metabolic abnormalities even while maintaining mice on a high-fat diet (27), demonstrating a causative role for the decrease in hepatic CEACAM1 level in sustaining diet-induced systemic insulin resistance and hepatic steatosis. That impairment of insulin clearance plays a significant role in hepatic insulin resistance in response to high-fat diet has recently been demonstrated in Asian men (28). Using a two-step hyperinsulinemic-euglycemic clamp, Bakker et al. (28) showed that in contrast to age- and sex-matched Caucasians, young and healthy South Asian men develop impairment of insulin clearance as well as hepatic insulin resistance in the absence of other metabolic alterations in skeletal muscle and white adipose tissue following 5 days of a high-fat Western diet intake. Several other studies in humans (28) as well as dogs (29) have supported the findings that defective hepatic insulin clearance is implicated in diet-induced insulin resistance.

The decrease in hepatic CEACAM1 by high-fat diet is attributed to lipolysis-derived FFA, in agreement with reducing hepatic CEACAM1 levels by intralipid–heparin infusion (24) and the negative effect of FFA on insulin clearance (30, 31). The underlying mechanism of CEACAM1 repression by FFA is *via* PPARα activation (4). In the presence of normoinsulinemia, this provides a positive feedback mechanism on fatty acid β-oxidation as it limits the negative effect of CEACAM1 on FASN activity (11) and subsequently, reduces malonyl-CoA-mediated inhibition of fatty acids translocation to the mitochondria (3). When CEACAM1 level is reduced by >50%, hepatic insulin clearance fails and chronic hyperinsulinemia develops, causing hepatic insulin resistance, at least in part by downregulating insulin receptors in the hepatocyte (32, 33) and triggering *de novo* lipogenesis by activating SREBP1c-mediated transcription of lipogenic genes (13), including acetyl-CoA carboxylase (ACC), a limiting enzyme in lipid biosynthesis. Elevation in ACC level (and activity) induces malonyl-CoA level, which in turn, inhibits fatty acid transport to the mitochondria and β-oxidation. Potentially contributing to the downregulation of β-oxidation under hyperinsulinemic conditions is the maintenance of insulin-stimulated phosphorylation and inactivation of Foxa2-mediated suppression of the transcription of genes involved in fatty acid β-oxidation (34, 35). Collectively, this limits fatty acid β-oxidation while promoting *de novo* lipogenesis, leading to hepatosteatosis. With the loss of the potential counter-regulatory anti-inflammatory function of CEACAM1, this causes a more robust change in the inflammatory milieu of the liver and steatohepatitis develops. Together, the data identify reduction in CEACAM1 expression as a novel molecular underpinning of the integrated regulation of lipid oxidation and hepatic insulin resistance (gluconeogenesis) by FFA mobilization from white adipose tissue (36–38).

## Reduced Hepatic CEACAM1 Levels Causes Obesity by Contributing to Energy Imbalance

High-fat diet represses hepatic CEACAM1 levels to impair insulin clearance and cause hyperinsulinemia that in turn, drives increased hepatic lipid production and output to the white adipose depot for storage (39). This is consistent with the well-accepted association of hyperinsulinemia and liver steatosis with high plasma Apolipoprotein B levels and visceral obesity in humans and rodents (40–45). Together with visceral obesity, sustained hyperinsulinemia reduces glucose transporter 4-mediated glucose transport to cause insulin resistance in adipose tissue (46), as supported by hyperinsulinemic-euglycemic clamp analysis in Ceacam1 mutants (16–18, 47) and in the diet-induced model (26).

Consistent with the finding that reduction of hepatic CEACAM1 plays a critical role in diet-induced altered metabolic response, transgenic protection of hepatic CEACAM1 in L-CC1 mice prevents hyperinsulinemia, insulin resistance, and hepatosteatosis in response to high-fat diet (26). It also limits the size of adipocytes and total fat mass by countering the negative effect of high-fat diet on energy expenditure and spontaneous physical activity (26). Similarly, adenoviral-redelivery of wild-type CEACAM1 in the liver protects energy balance against high-fat intake, thereby reversing the gain in body weight and visceral adiposity (27). Given that CEACAM1 is not detected in the adipocyte at the protein level (2), it is likely that the gain-of-function of hepatic CEACAM1 drives this positive effect on energy expenditure and adipose tissue biology (limited adipocyte size, fibrosis, and inflammation) (27, 39). The beneficial effect of hepatic CEACAM1 gain-of-function on insulin response in white adipose tissue could be mediated, at least in part, by the rise in plasma FGF21 (48, 49) that induces the locomotor activity (50) and energy expenditure (51, 52).

Both L-SACC1 and *Cc1*^−/−^ mutant mice display visceral obesity and a higher body mass than their wild-type counterparts (16–18). Visceral obesity, which is partly caused by elevated hepatic lipid production and redistribution to white adipose tissue (19), leads to hyperleptinemia, which could in turn, alter response to leptin and cause energy imbalance. Consistently, global *Cc1*^−/−^ null mice develop elevated production and secretion of leptin from their expanded while adipose depot in addition to increased total fat mass and obesity resulting from hyperphagia and reduced spontaneous physical activity (53). In addition to leptin resistance, hyperinsulinemia also contributes to the obesity phenotype in these mice, at least in part, by inducing hypothalamic FASN level and activity (53), which in turn, causes hyperphagia (54) and lower physical activity (55, 56). Together, this demonstrates that altered CEACAM1-dependent insulin clearance pathways drive hyperinsulinemia-mediated link of hepatic steatosis to visceral obesity and increased total fat mass.

## Concluding Remarks

**Figure 1:**
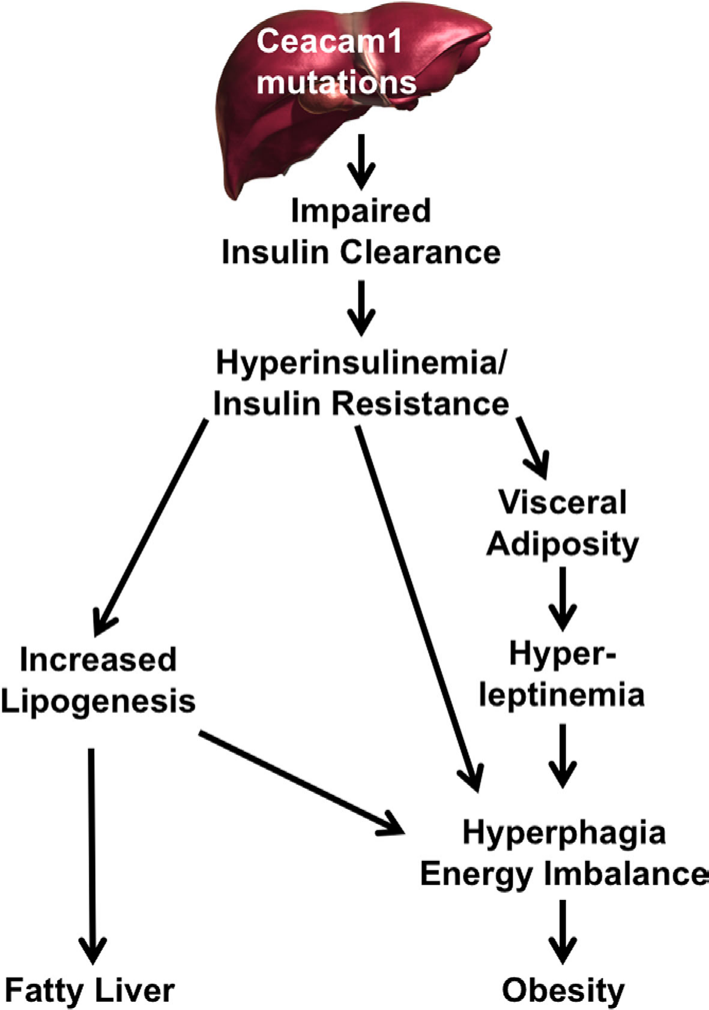
**A pivotal role for carcinoembryonic antigen-related cell adhesion molecule 1 (CEACAM1) reduction in the pathogenesis of fatty liver disease and obesity**. Reduction or mutation of Ceacam1 in the liver results in decreased insulin clearance from the portal circulation. Reduced clearance leads to hyperinsulinemia followed by insulin resistance (owing to downregulation of the insulin receptor) and increased hepatic lipogenesis. Elevation in hepatic lipogenesis leads to lipid redistribution to the while adipose depot to increase visceral adiposity. This leads to hyperleptinemia, which along with hyperinsulinemia, increases food intake and energy imbalance, further exacerbating obesity. Hyperinsulinemia drives hepatic lipogenesis and fat accumulation in liver.

The mechanisms underlying the pathogenesis of non-alcoholic fatty liver disease (NAFLD) in humans remain unclear (57) and whether insulin resistance plays a role in NAFLD has been debated, owing to the lack of appropriate animal models that replicate all features of the human disease and its progression to NASH (58, 59). As summarized in this review, our laboratory has demonstrated in the last couple of decades that loss in hepatic CEACAM1 expression and its defective phosphorylation impair insulin clearance and subsequently, play a pivotal role in insulin resistance, fatty liver disease, and obesity (Figure 1) (9, 10, 16–19, 25, 27, 39, 53, 60, 61). Demonstration of a role for impaired insulin clearance in insulin resistance in human disease is emerging (62–65). In this regard, compromised hepatic insulin extraction has been shown to constitute a risk factor for obesity (66, 67), type 2 diabetes (68), metabolic syndrome (65, 69), and fatty liver disease (70). The study by Lee (71) showing a marked decline in hepatic CEACAM1 levels in patients with high-grade fatty liver and obesity coupled with our mechanistic studies demonstrating that redelivering CEACAM1 to the liver reverses diet-induced insulin resistance, fatty liver, and visceral obesity (27) emphasizes a critical role for CEACAM1 in metabolic control. Of note, while our studies show that reduction of hepatic CEACAM1 causes insulin resistance, hepatosteatosis, and visceral obesity, they also show that diet-induced visceral obesity represses hepatic CEACAM1 to cause fat accumulation in liver and insulin resistance (3, 26, 27). Further emphasizing the metabolic role of hepatic CEACAM1, liver-specific overexpression of CEACAM1 curbs the metabolic abnormalities caused by high-fat diet and prevents insulin resistance and hepatosteatosis (26). Similarly, adenoviral-mediated redelivery of CEACAM1 to the liver reverses diet-induced metabolic derangement (27). Collectively, this positions the loss of hepatic CEACAM1 expression (and its resulting hyperinsulinemia and insulin resistance) on the crossroad of the pathogenesis of NAFLD and obesity.

## Author Contributions

GH wrote a first draft of the manuscript. HG, SG, HM, KR, QA-S, TB, DZ contributed to the writing. RG and LY reviewed the manuscript. SN was responsible for revising the manuscript. SN had full access to all the data of the study and takes responsibility for the integrity and accuracy of data analysis and the decision to submit and publish the manuscript.

## Conflict of Interest Statement

The authors declare that the research was conducted in the absence of any commercial or financial relationships that could be construed as a potential conflict of interest.
